# Differential Bacterial Community of Bee Bread and Bee Pollen Revealed by 16s rRNA High-Throughput Sequencing

**DOI:** 10.3390/insects13100863

**Published:** 2022-09-23

**Authors:** Sampat Ghosh, Saeed Mohamadzade Namin, Chuleui Jung

**Affiliations:** 1 Agriculture Science and Technology Research Institute, Andong National University, Andong 36729, Korea; 2 Department of Plant Protection, Faculty of Agriculture, Varamin-Pishva Branch, Islamic Azad University, Varamin 3381774895, Iran; 3 Department of Plant Medicals, Andong National University, Andong 36729, Korea

**Keywords:** nutrition provisioning, *Apis mellifera*, probiotics, health, metabolism, metagenomics, microbial ecology

## Abstract

**Simple Summary:**

Gut symbionts play a crucial role in the nutrition provisioning of honeybees. However, the data on the bacterial communities of pollen collected by bees and bee bread are comparatively scarce. Therefore, the present study was designed to explore and identify the bacterial communities from them. The study reports that the bacterial diversity was significantly higher in the bee bread than in bee pollen. The higher bacterial diversity in the bee bread could presumably be due to factors such as storage period, processing of food, fermentation, and high sugar environment. The bacterial communities of bee bread possibly exhibit beneficial roles for honeybees such as polysaccharide degradation and nitrogen fixing.

**Abstract:**

We investigated the bacterial community of bee bread and bee pollen samples using an approach through 16 s rRNA high-throughput sequencing. The results revealed a higher bacterial diversity in bee bread than in bee pollen as depicted in taxonomic profiling, as well as diversity indices such as the Shannon diversity index (3.7 to 4.8 for bee bread and 1.1 to 1.7 for bee pollen samples) and Simpson’s index (>0.9 for bee bread and 0.4–0.5 for bee pollen). Principal component analysis showed a distinct difference in bacterial communities. The higher bacterial diversity in the bee bread than bee pollen could presumably be due to factors such as storage period, processing of food, fermentation, and high sugar environment. However, no effect of the feed (rapeseed or oak pollen patties or even natural inflow) was indicated on the bacterial composition of bee bread, presumably because of the lack of restriction of foraged pollen inflow in the hive. The diverse bacterial profile of the bee bread could contribute to the nutritional provisioning as well as enhance the detoxification process; however, a thorough investigation of the functional role of individual bacteria genera remains a task for future studies.

## 1. Introduction

Honeybees forage for nectar and pollen. Nectar serves as a source of carbohydrates, and pollen is the primary source of protein, lipid, sterols, and micronutrients (minerals and vitamins). The nutritional ecology of bees including managed honeybees and wild bees provides insights into the plant–pollinator interaction and coevolution, and helps in understanding their foraging behavior and their food preference [1,2]. As pollen is the primary source of protein and amino acids essential for bee health, several studies have been conducted in connection with the protein content, quality of bee pollen, and foraging behavior of honeybees [3,4,5,6,7,8]. Our previous study conducted on foraging behavior and the preference of pollen sources indicated that the criteria of foraging preference would be nutritional contents such as protein [9]. Increasing the protein and amino acid concentration of the pollen diet improved honeybee health including immunocompetence [10,11]. Although the protein content and amino acid composition are undoubtedly important for bee health, fat and sterols have also emerged as influencing nutritional factors for bee foraging and health. Therefore, the protein: lipid ratio could be a guide for understanding the pattern of bee foraging and floral preference [1,12].

Honeybee foragers do not consume fresh pollen; they collect and bring pollen with corbiculae (bee-collected fresh pollen, hereafter denoted as bee pollen) into the hive. Further, the bee pollen is mixed with glandular secretions, nectar or honey, processed by symbiont microbes, and becomes bee bread, which nurse bees consume [13,14]. It is a common practice to provide pollen patties as a source of nourishment to the managed colonies of the apiary to overcome the nutritional deficiency of the colony. When beekeepers create pollen patties from bee pollen, changes in nutrient composition such as an increase in the amount of carbohydrates and protein and a decrease in lipid occur [15]. An increase in carbohydrate may be expected, which advantageous for bees as carbohydrate meets the energetic expenses, especially for the adult workers. Further, the process of bee bread preparation by honeybees in the hive leads to a temporal compositional transition of bee pollen to bee bread [13]. Microorganisms help in the processing of bee pollen to bee bread [14]. Symbiont microbes primarily facilitate nutrient provisioning mainly by fermentation and protective functions for honeybees. Environmental factors such as antibiotics, pesticides, diet, and season can alter the honeybee gut microbial composition, which might have possible consequences on metabolism, immune functions, detoxification, pathogen resistance, etc. Therefore, environmental factors could affect the fitness of honeybees. Several studies have been conducted to investigate the gut microbes of honeybees and have identified the core microbiome including core bacterial species that are implicated in the nutrition and development of workers [16,17,18,19,20,21,22]. Gut microbes, especially bacteria, play a critical role in the metabolism of carbohydrates present on the cell wall of pollen and also in fermentation acting as probiotics [23]. To cite examples, *Gilliamella apicola* can utilize monosaccharides such as mannose, arabinose, xylose, or rhamnose, which can be toxic to bees [24]. On the other hand, *Snodgrassella alvi* is a nonsugar fermenter [25]. Pesticide and herbicide exposure often perturbs the gut microbiota of honeybees. For example, glyphosate exposure disturbed the beneficial gut bacteria of honeybees such as *Snodgrassella alvi* and *G. apicola*, and this could potentially affect bee health [21,26,27]. In order to detoxify the pesticides and herbicides, the honeybee possesses genes related to the detox mechanism as well as the immune system [28]. In addition, microbiota could contribute to this detoxification process by degrading the xenobiotics including pesticides [29].

As bee breads are stored in the comb, which has different abiotic conditions than the outside [30], it might favor a different bacterial population than the bee pollen. Scientific attention has been focused largely in order to investigate the gut microbiome of honeybees and, on the other hand, there are comparatively less data on bee bread or bee pollen. A few studies have been conducted on the microbial ecology of the hive including bee bread, and most of them have been conducted in the United States of America and Europe [31,32,33,34,35]. Landscape has an influence on the food resource of honeybees and the associated microbial dynamics, and hardly any studies have been carried out in the Korean context. Therefore, the present pilot study has been undertaken to investigate the bacterial communities of beebread and bee pollen. Further, we examined the effect of feed (pollen patties made up of different bee pollens) on the bacterial communities of the bee bread.

## 2. Materials and Methods

### 2.1. Study Design

Honeybee *Apis mellifera ligustica* queen-right colonies from the experimental apiary at Andong National University were recruited in spring (during the end of February and March 2020) to carry out the investigation of the microbiome of bee bread and bee pollen. Seven healthy honeybee hives of similar strength and composition were maintained and used in this study. Three hives marked A were provisioned with patties made from oak pollen and another three were marked R and provisioned with patties made from rapeseed pollen. The seventh hive was used as the control. One among the A and one among R hives were installed with a pollen trap and marked AT and RT, respectively. Samples of at least 20 g of bee bread were collected from each hive on the 5th day, removed using sterile tweezers and needles and brought to the laboratory, and stored at −20 °C until further processing. The samples collected from each hive were well mixed and a composite sampling method was followed for the analyses. Similarly, bee pollen samples were collected from the two hives, namely AT and RT (P1 and P2, respectively), which were equipped with pollen traps and stored. As the honeybee feeding regime is <72 h [35], we expected that once the pollen patty was installed in the hive, 5 days would be sufficient to completely consume the already stored food for honeybees in the hive and to influence the bee bread preparation.

### 2.2. DNA Extraction and Sequencing

Microbial DNA extraction from bee bread (250 mg) and bee pollen (250 mg) was carried out using the QIAGEN DNeasy PowerSoil Kit (QIAGEN, Hilden, Germany) according to the manufacturer’s instructions. For each DNA sample, the concentration of nucleic acid was evaluated by a Life Real spectrophotometer (Bioer Technology Co., Ltd., Zhejiang, China). Extracted DNA samples were kept at −20 °C prior to library preparation. In order to investigate microbial diversity and structure comparisons from bee bread as well as bee pollen, the V3–V4 region of 16S rRNA was amplified using the primers 341F (5′-CCTACGGGNGGCWGCAG-3′) and 805R (5′-GACTACHVGGGTATCTAATCC-3′) and sequenced with an Illumina MiSeq (Macrogen, Seoul, Korea). The sequences were submitted to NCBI through the project number PRJNA826229.

### 2.3. Sequence Analysis

The quality of raw paired-end reads was initially assessed using FastQC (Babraham Bioinformatics, Cambridge, UK). The raw sequences were imported into Qiime2 [36] and the analysis of the sequences was conducted using the DADA2 algorithm, which included trimming of the length of forward reads to 280 bp and reverse reads to 220 bp to obtain high-quality reads (a Phred quality score of at least 20), assembly of forward and reverse reads, and chimera detection. The Amplicon Sequence Variants (ASVs) were classified by default against the SILVA v132 database to assign taxonomy. The evolutionary roots of chloroplast and mitochondria are cyanobacteria and rickettsiales, respectively [37,38], and, therefore, in order to avoid contamination with higher plant taxa, all the chloroplastidial and mitochondrial sequences were removed.

### 2.4. Biodiversity Indices Measurement and Statistical Analysis

Alpha-diversity values of the bee bread and bee pollen samples, i.e., Shannon diversity index (H’) and Simpson’s index (1-D), were calculated by computing read count values in Excel.

$$\mathrm{Shannon}\;\mathrm{Diversity}\;\mathrm{Index}\;(H’)=-\sum_{i=1}^{s}\left(p_{i}\ln p_{i}\right)$$

In the Shannon index, p is the proportion of read counts of a sequence representing a bacterial genus (i-th) divided by total read counts of all bacterial genera of a sample.

$$\mathrm{Simpson}\;\mathrm{Index}\;(1-D)=1-[\sum n_{i}\left(n_{i}-1\right)/N\left(N-1\right)]$$

In the Simpson’s index, n_i_ is the read counts of a sequence representing a bacterial genus (i-th) and N is the total read counts of all bacterial genera present in a sample.

The difference in biodiversity indices between bee bread and bee pollen was measured by the *t*-test in SPSS 16.0 (IBM, New York, NY, USA). Principal Component Analysis (PCA) among the bee bread and bee pollen samples, and the correlation among the predominant bacterial genus were carried out using R software and the *gg plot* package [39]. The biochemical properties of bacterial genera were obtained from Bergey’s manual of Systematic Bacteriology [40].

## 3. Results

**Figure 1:**
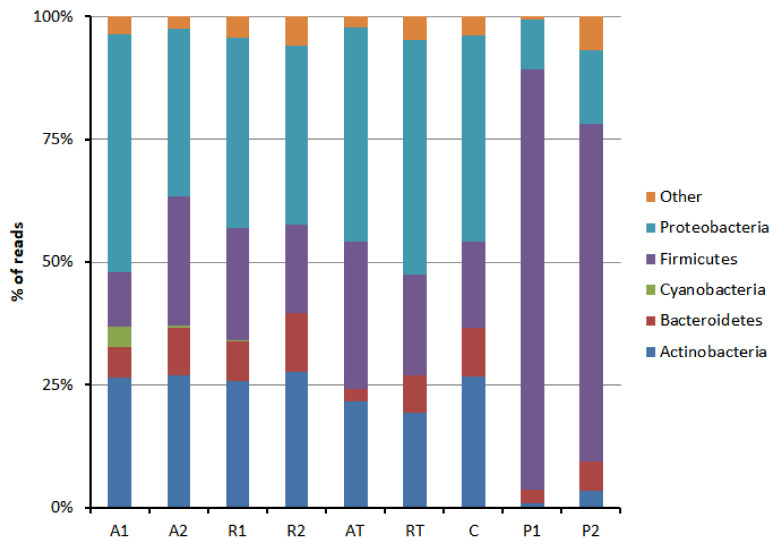
Phylum-wise distribution pattern of bacterial population in bee bread and bee pollen samples (A1 and A2 = bee breads collected from two colonies fed with oak pollen patty; R1 and R2 = bee breads collected from two colonies fed with rapeseed pollen patty; AT = bee bread collected from colony fed on oak pollen patty with restricted inflow of pollen from outside; RT = bee bread collected from colony fed on rapeseed pollen patty with restricted inflow of pollen from outside; P1 and P2 = bee pollen collected from bee hives equipped with pollen trap; C = bee bread collected from control hive).

**Figure 2:**
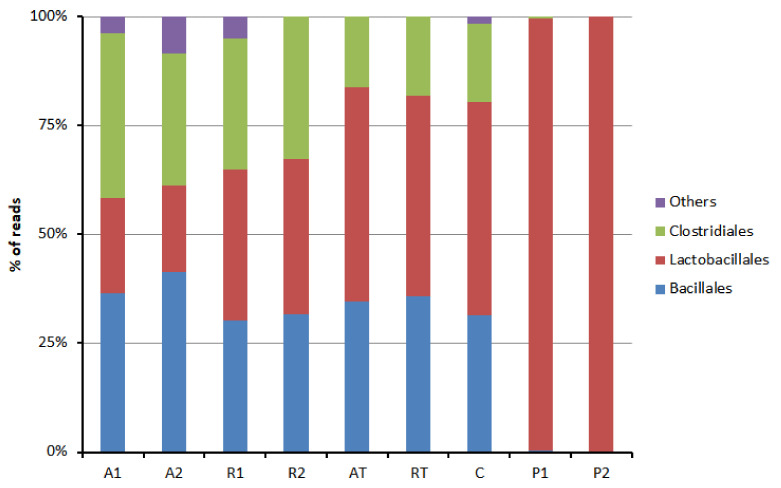
Order-wise distribution of phylum Firmicutes in bee bread and bee pollen samples (A1 and A2 = bee breads collected from two colonies fed with oak pollen patty; R1 and R2 = bee breads collected from two colonies fed with rapeseed pollen patty; AT = bee bread collected from colony fed on oak pollen patty with restricted inflow of pollen from outside; RT = bee bread collected from colony fed on rapeseed pollen patty with restricted inflow of pollen from outside; P1 and P2 = bee pollen collected from bee hives equipped with pollen trap; C = bee bread collected from control hive).

The phylum-wise distribution of the bacterial population is represented in Figure 1. Bee bread exhibited a higher bacterial diversity in the bee pollen. The majority of the bacterial taxonomic unit (>95%) was found belonging to five phyla, namely Actinobacteria, Bacteroidetes, Cyanobacteria, Firmicutes, and Proteobacteria. Others belonged to phyla Acidobacteria, Armatimonadetes, Chloroflexi, Deinococcus-Thermus, Dependentiae, Epsilonbacteraeota, Fusobacteria, Gemmatimonadates, Nitrospira, Patescibacteria, Planctomycetes, Tenericutes, and Verrucomicrobia. Firmicutes were the most predominant phyla found for bee pollen. In contrast, Proteobacteria was abundant in bee bread followed by Firmicutes. An order-wise distribution of Firmicutes was found in bee pollen and bee bread, as shown in Figure 2. The majority of Firmicutes were represented by orders bacillales, lactobacillales, and clostridiales in bee bread and by only lactobacillales in bee pollen (Figure 2). Two major orders belonging to Bacteroidetes were found flavobacteriales and sphingobacteriales, both of which are typical soil bacteria.

**Figure 3:**
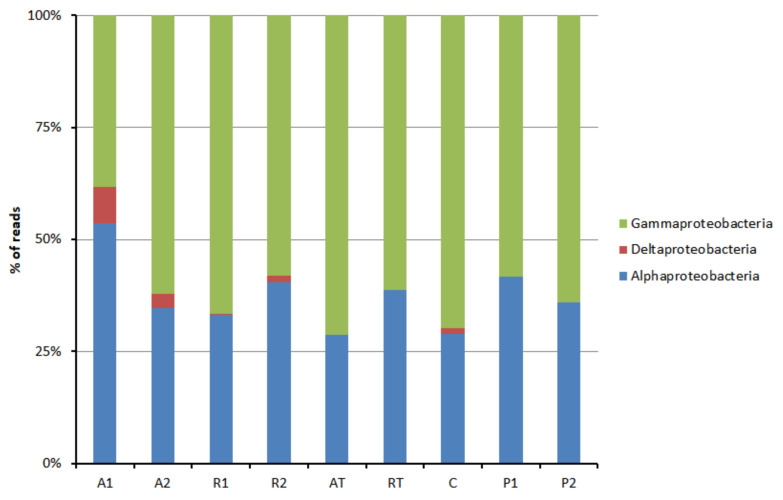
Class-wise distribution of phylum Proteobacteria in bee bread and bee pollen samples (A1 and A2 = bee breads collected from two colonies fed with oak pollen patty; R1 and R2 = bee breads collected from two colonies fed with rapeseed pollen patty; AT = bee bread collected from colony fed on oak pollen patty with restricted inflow of pollen from outside; RT = bee bread collected from colony fed on rapeseed pollen patty with restricted inflow of pollen from outside; P1 and P2 = bee pollen collected from bee hives equipped with pollen trap; C = bee bread collected from control hive).

**Figure 4:**
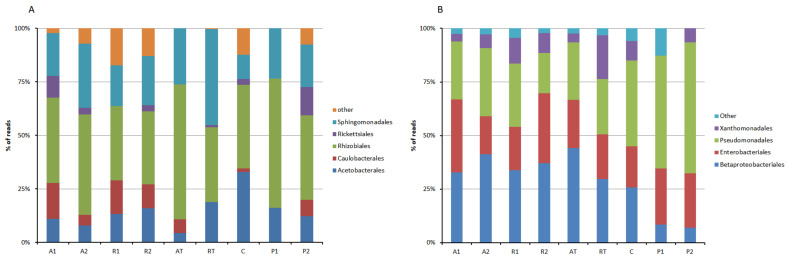
Order-wise distribution of Alpha-Proteobacteria (**A**) and Beta-Proteobacteria (**B**) in bee bread and bee pollen samples (A1 and A2 = bee breads collected from two colonies fed with oak pollen patty; R1 and R2 = bee breads collected from two colonies fed with rapeseed pollen patty; AT = bee bread collected from colony fed on oak pollen patty with restricted inflow of pollen from outside; RT = bee bread collected from colony fed on rapeseed pollen patty with restricted inflow of pollen from outside; P1 and P2 = bee pollen collected from bee hives equipped with pollen trap; C = bee bread collected from control hive).

The distribution of the bacterial population belonging to the phyla Proteobacteria is depicted in Figure 3. Bee breads collected from nonrestricted beehives were found to contain Proteobacteria, which belong to three classes, namely alpha-proteobacteria, delta-proteobacteria, and gamma-proteobacteria. However, no delta-proteobacteria was found in bee pollen, as well as bee breads collected from beehives with a pollen trap installed. In this context, it is worth mentioning that although beta-proteobacteria is a class of phylum Proteobacteria, SILVA 132 assigned them as (beta-proteobacteriales), an order under class gamma-proteobacteria. The evolutionary root of mitochondria falls within rickettsiales and, therefore, the scope of contamination with a genome other than bacteria exists. To avoid contamination, the sequences identified as mitochondria were removed from rickettsiales. After removing the sequences identified as mitochondria, among alpha-proteobacteria, rhizobiales were found the most abundant (Figure 4A). Beta-proteobacteriales, enterobacteriales, and pseudomonadales are major orders found under gamma-proteobacteria (Figure 4B).

**Figure 5:**
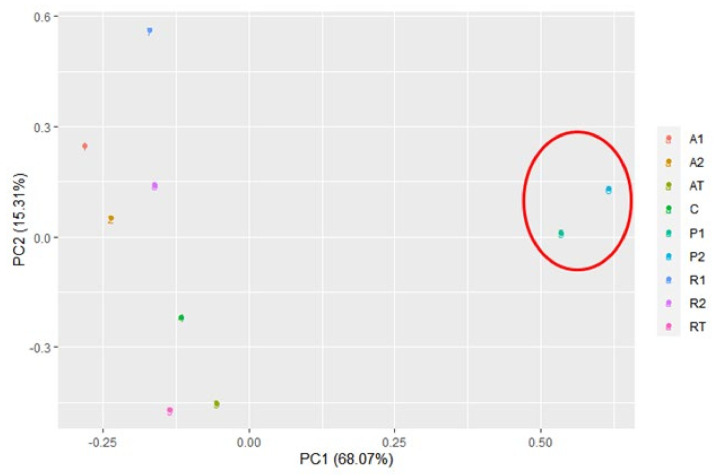
Principal Component Analysis (PCA) of bee bread and bee pollen samples based on major bacterial genera (A1 and A2 = bee breads collected from two colonies fed with oak pollen patty; R1 and R2 = bee breads collected from two colonies fed with rapeseed pollen patty; AT = bee bread collected from colony fed on oak pollen patty with restricted inflow of pollen from outside; RT = bee bread collected from colony fed on rapeseed pollen patty with restricted inflow of pollen from outside; P1 and P2 = bee pollen collected from bee hives equipped with pollen trap; C = bee bread collected from control hive). The red circle clearly indicated the clustering of bee pollen.

Quantitatively, the bacterial diversity was represented by the Shannon diversity index and Simpson’s index, as represented by Table 1. The Shannon diversity index was found within the range of 3.7 to 4.8 for bee bread samples and was significantly higher than that of bee pollen, accounting for 1.1 and 1.7 (*p* < 0.05). Similarly, Simpson’s index (1-D) was also found to be higher for bee bread samples (>0.9) than for bee pollen (0.4, 0.5). Both of these indices reflected the higher species diversity and relative abundance in bee bread. However, although the metabarcoding revealed 440 bacterial genera, 25 were the most predominant based on their read counts. The predominant genera along with their biochemical characteristics are represented in Table 2. In contrast to bee bread, beta-proteobacteriales were found significantly less in bee pollen. PCA analysis for the predominant bacterial genus demonstrated the separation of clusters for bee pollen and bee bread (Figure 5). However, no clear clusters were indicated for bee bread obtained from hives fed on different feeds.

## 4. Discussion

Although honeybees are considered as generalist pollinators, actual resource use is represented by a comparatively small number of core species of plants [41]. An investigation on honeybee foraging in the National Botanic Garden of Wales during Spring (April and May) revealed that out of 437 plants genera, honeybees used only 39 plants and, among them, only 10 plants were core-used plant species as revealed from honey [42]. Compared with different bumblebee species, the foraging of honeybee *Apis mellifera* is more flower-constant in a particular landscape [7]. One of the major components of the nutritional ecology of honeybees is their foraging decision-making process, which presumably depends on the nutrient requirement of the colony, i.e., energy balance of the colony. Foragers gather pollen and nectar to provide the required nutrition and to sustain the optimal colony development [43]. Therefore, honeybee colonies of similar strength and composition could expect a similar foraging behavior, food preference, and consistent microbial population.

The higher bacterial diversity in the bee bread in comparison to bee pollen, as depicted in this study, was presumably due to the processing of bee bread, which involves fermentation and different environmental conditions such as maintained temperature, higher humidity, anaerobic condition, and high sugar environment in the beehive. These abiotic conditions might be favorable for different bacterial populations found on the anthers. Honeybees maintain microclimatic conditions within their beehives. The normal range of temperature within a honeybee colony is reported within the range of 33 to 36 °C [30,44], which is favorable to most of the bacterial population. They also maintain relative humidity (RH) as a RH lower than 50% inhibits the hatching of eggs and a RH of 90–95% is optimum for egg hatching (reviewed by Abou-Shaara et al. [30]). In addition to these abiotic conditions, the preparation of bee bread involving glandular secretions and mixing of bee pollen and nectar [13] could contribute the microbial load to bee bread. Species richness and diversity were found highest in *Osmia cornuta* bee bread than bee pollen [34], which has a similar trend with the present study. The study moreover demonstrated that the old bee bread was found to have more diversity and species richness than the fresh bee bread [34]. However, Anderson et al. [35] demonstrated that honeybee bread older than 96 h had a significantly declined bacterial population. Higher bacterial diversity was found higher in honeybee bread and brood than the bee gut [33].

Contamination of soil bacteria could be possible as the hives were located in close proximity of the ground. Some of the soil bacteria were found to be efficient at degrading the xenobiotics such as pesticides and could be beneficial for honeybees [29]. However, the scope of examining the bioremediation of the microbial population was beyond the scope of the present study.

Microbial communities associated with nutrition provisioning, mainly present in the ileum, and bee health presumably co-evolved with food transfer, storage, digestion, and processes in the enzymatically active and nutrient-rich midgut [23,45]. In addition to the function of the ileum in water and nutrient absorption [46], the presence of symbionts, particularly *Snodgrassella alvi* and *Gilliamella apicola*, is involved in the biofilm formation, which presumably provides a protective layer against parasites [47]. Some bacterial communities are present in the rectum, generally provided with unused nutrients during winter [45]. Generally, the honeybee gut bacteria belong to three major phyla such as Firmicutes, Proteobacteria, and Actinobacteria, among which Firmicutes are predominant [23]. A study by Corby-Harris et al. demonstrated that despite a very different diet, the forager honeybee gut contains core microbiota similar to that found in the gut of younger honeybees [19]. Similar to the honeybee gut microbiota, Firmicutes were found to be the most abundant in the bee pollen. Firmicutes and bacteroidetes are polysaccharide degraders [48]. Lactobacillus represented the most abundant bacteria belonging to Lactobacillales. The Gram-positive, catalase-negative, non-spore-forming bacteria produce lactic acid as the major end product of their fermentation process. However, they may be categorized on the basis of their three distinct carbohydrate fermentation pathways, i.e., obligate homo-fermentative, facultative hetero-fermentative, and obligate hetero-fermentative [49]. Evans and Lopez demonstrated that nonpathogenic bacteria *Bifidobacterium* and *Lactobacillus* can be used as probiotics to enhance honeybee immunity [50]. In addition, several scientific reports showed the potential of using *Lactobacillus* and *Bacillus* as probiotics to stimulate queen egg-laying, improve health status, and increase the size of wax cells, etc. [51].

Among the Proteobacteria, gamma-proteobacteria was predominant followed by alpha-proteobacteria in all the samples of bee bread and bee pollen. This is in agreement with the gut bacterial composition of honeybees, where gamma-proteobacteria was also abundant in the phyla Proteobacteria [23]. Gamma-proteobacteria was also found predominant, followed by alpha-proteobacteria in most of the bee breads studied in the apiary sites located in northwest England except a few where the reverse was true [31]. In the present study, *Massilia*, *Duganella*, *Comamonas*, etc., were found major among beta-proteobacteriales. Beta-proteobacteriales are known for their functionalities such as nitrogen fixation, oxidizing ammonia into nitrites, which could presumably enrich the proteinaceous nutritional turnover of bee bread. On the other hand, rhizobiales, a well-studied order of alpha-proteobacteria, exert beneficial roles for their host plant by providing nutrients, phytohormones, and necessary precursors of plant metabolites [52]. The order contains nitrogen-fixing, methanotrophic, legume-modulating, micro-symbiotic bacteria [52,53]. In the present study on bee bread and bee pollen, the most abundant family under Rhizobiales found was Beijerinckiaceae followed by Rhizobiaceae. The most predominant genus found was *Methylobacterium*, which was found in all the bee bread and bee pollen. However, in the study on the bacterial communities of bee bread from Carl Hayden Bee Research Center in Tucson America, it was found that the most abundant bacteria was Firmicutes, especially *Lactobacillus* [32].

Acidobacteria play a vital role in the carbon cycle and they are capable of degrading lignin and cellulose [54], which is a component of the cell wall of plant cells including pollen; therefore, they might benefit honeybees in breaking the pollen cell wall. On the other hand, actinobacteria are widely distributed in terrestrial ecosystems mainly in soil. In Actinobacteria, the most predominant order found was corynebacteriales. Micrococcales, propionibacteriales, and streposporangiales were also found in abundance. Bacteria belonging to cornybacteriales might enhance the level of amino acids such as glutamic acid, lysine, and histidine in the bee bread as the bacteria conduct a fermentative process and produce the amino acids [55]. Propionic acid is a primary metabolite of propionibacteriales, which acts as a mold inhibitor, and propionibacteria also synthesize vitamin B12 [56] which might enrich the bee bread and is essential for honeybee brood production [57].

A significant portion of cyanobacteria was identified as ASVs belonging to the chloroplast. Cyanobacteria are the ancestor of chloroplast and, therefore, the ASVs were assigned to the cyanobacteria phylum. In the case of identifying them up to the species level, it was found that several were of plant origin and did not belong to the bacteria, and many were unidentified. Based on the predominant bacterial genus, PCA analysis clearly demonstrated separate clusters for bee pollen and bee bread. However, no clear clusters were found for bee bread obtained from different hives fed on a different feed. In this regard, it is noteworthy that the hives fed on different feeds were not restricted by the inflow of the bee pollen foraged by the foragers. Presumably, mixing the bee pollen with the feed provided could have masked the distribution of the clusters.

## 5. Conclusions

The study revealed a clear difference of the bacterial community composition in bee pollen and bee bread. Bee bread had more diverse and rich bacterial communities than those of bee pollen, which is presumably because of the different abiotic conditions of bee bread than pollen, the addition with secretions, fermentation, and the feeding behavior of honeybees. However, no effect of the feed (rapeseed or oak pollen patties or even natural inflow) was identified on the bacterial composition of bee bread, indicating that, regardless of the source of bee pollen, honeybees interact with environmental sources of bacteria to formulate and manipulate the food source, bee bread. Investigation of the functional role of individual bacteria genera, found in the study, on the nutrition provisioning for honeybees remains a task for future studies.

## Figures and Tables

**Table 1:** Bacterial biodiversity indices of the studied bee bread and bee pollen (A1 and A2 = bee breads collected from two colonies fed with oak pollen patty; R1 and R2 = bee breads collected from two colonies fed with rapeseed pollen patty; AT = bee bread collected from colony fed on oak pollen patty with restricted inflow of pollen from outside; RT = bee bread collected from colony fed on rapeseed pollen patty with restricted inflow of pollen from outside; P1 and P2 = bee pollen collected from bee hives equipped with pollen trap; C = bee bread collected from control hive).

Treatment	Hive	Simpson’s Index	Shannon Diversity Index
Provisioned with oak pollen patty	A1	0.978657	4.36757
	A2	0.987332	4.759792
Provisioned with rapeseed pollen patty	R1	0.986811	4.655952
	R2	0.987809	4.712931
Provisioned with oak pollen patty and trap installed	AT	0.963492	3.669413
Provisioned with rapeseed pollen patty and trap installed	RT	0.976405	4.091421
Control	C	0.982049	4.428812
Bee pollen from AT hive	P1	0.386309	1.132402
Bee pollen from RT hive	P2	0.534527	1.748493

**Table 2:** Major bacterial genera found in the bee bread and bee pollen and their biochemical characterization in most of the cases of the genus (biochemical properties of bacterial genera were obtained from Bergey’s manual of Systematic Bacteriology).

Phylum	Genus	Characterization			
		Gram Staining	Cellular Respiration	Catalase	Oxidase
Actinobacteria	Corynebacterium	+ve	Aerobic (most are)	+ve	−ve (except a few)
	Mycobacterium	Acid fast, +ve	Aerobic	+ve	−ve
	Nocardioides	+ve	Aerobic	+ve/−ve	−ve
	Streptomyces	+ve	Aerobic	+ve	−ve
	Actinomadura	+ve	Aerobic	+ve	−ve
Bacteroidetes	Flavobacterium	−ve	Aerobic	+ve/−ve	+ve
	Chryseobacterium	−ve	Aerobic	+ve	+ve
	Sphingobacterium	−ve	Aerobic	+ve	+ve
Firmicutes	Bacillus	+ve	Aerobic, (under some conditions) anaerobic	+ve	variable
	Paenibacillus	+ve, −ve, variable	Facultative anaerobic/strictly aerobic	+ve	+ve
	Lactobacillus	+ve	Aerotolerant anaerobes or microphilic	−ve	−ve
	Streptococcus	+ve	Facultative anaerobic	−ve	−ve
	Peptoniphilus	+ve	Anaerobic	−ve	−ve
Proteobacteria	Methylobacterium	−ve	Aerobic	+ve	+ve
	Shingomonas	−ve	Strictly aerobic	+ve	+ve
	Comamonas	−ve	Aerobic	+ve	+ve
	Duganella	−ve	Aerobic	+ve	+ve
	Massilia	−ve	Aerobic	+ve	+ve
	Enterobacter	−ve	Facultative anaerobic	+ve	−ve
	Pantoea	−ve	Facultative anaerobic	+ve	−ve
	Acinetobacter	−ve	Strictly aerobic	+ve	−ve
	Psychrobacter	−ve	Aerobic	+ve	+ve
	Pseudomonas	−ve	Aerobic	+ve	−ve
	Pseudoxanthomonas	−ve	Aerobic	+ve	+ve
	Stenotrophomonas	−ve	Aerobic	+ve	+ve/−ve

## Data Availability

Available online: https://dataview.ncbi.nlm.nih.gov/object/PRJNA826229?reviewer=48h25rkfou21vc5f1bcki22saj (accessed on 31 May 2022).
